# Geographic divergence of “*Sulfolobus islandicus”* strains assessed by genomic analyses including electronic DNA hybridization confirms they are geovars

**DOI:** 10.1007/s10482-013-0081-4

**Published:** 2013-12-04

**Authors:** Guanghong Zuo, Bailin Hao, James T. Staley

**Affiliations:** 1 T-Life Research Center, Fudan University, Shanghai, 200433 China; 2 Department of Microbiology, University of Washington, Seattle, WA 98195 USA

**Keywords:** Prokaryote species concept, Archaea biogeography, *Sulfolobus islandicus* geovars, In silico DNA hybridization, Average nucleotide identity, Whole-genome CVTree phylogeny

## Abstract

Ten well-annotated genomes of “*Sulfolobus islandicus*” strains from different geographic locations have been released at the NCBI database. Whole genome based composition vector trees indicate that these strains show the same branching patterns as originally reported by multi-locus sequence analysis. To determine whether the ten strains meet the criteria for separate species, DNA–DNA hybridization (DDH) was performed in silico. DDH values of strains from the same geographic location, i.e., Iceland, Kamchatka and North America, ranged from 82.4 to 95.4 %, clearly qualifying them as members of the same species. The lowest DDH values found between locations ranged from 75.5 to 76.6 %, which exceed the 70 % DDH threshold for a species thereby indicating they are all members of the same species based on the currently accepted definition. The clear divergences of strains from the different geographic locations are sufficiently great to consider them as separate geovars. “*S. islandicus*” has not yet been validly named and a type strain has not been deposited in culture collections. We urgently recommend that those who study the organism fulfill the criteria of the International Code of Nomenclature of Bacteria in order to designate a type strain and to identify and deposit related strains of this species to make them available to the broader scientific community.

## Introduction

Biogeography plays an extremely important role in the speciation of plants and animals. Allopatric speciation occurs when plant or animal species are geographically separated from one another over a long period of time (Staley 2004). For example, islands such as the Hawaii Islands in the Pacific Ocean are separated from other land masses by thousands of kilometers. Those species that managed to be transported there have speciated over millions of years to form novel species, primarily through genetic drift and selection.

Although geography plays a major role in plant and animal speciation, its role in bacterial and archaeal speciation is poorly understood. Indeed, at this time there is only one reported species of prokaryotic organism, the thermophilic, acidophilic archaeon, “*Sulfolobus islandicus”,* in which biogeography has been shown to play a role in speciation (Whitaker et al. 2003).

At this time it is not yet known whether sufficient speciation has occurred among the strains from different locations to justify separate species for the geographic varieties that have been reported. In order to determine whether these are separate species of prokaryotes, it is necessary to carry out DNA–DNA hybridization (DDH) among the strains. Genomes are now available of ten strains of “*S. islandicus”* that have been isolated from four separate hot spring locations (Iceland; Yellowstone National Park, WY and Lassen Park, CA in North America; and Kamchatka in Russia). In this paper we performed DDH using in silico (computational) procedures (Auch et al. 2010a, b; DSMZ 2013) to determine whether the strains are sufficiently divergent to warrant separate species status. According to the most widely accepted bacterial species definition (Brenner et al. 2005; Staley 2006; Wayne 1987), a value of more than 70 % DDH is necessary between any of the strains to qualify them as members of the same species.

Unfortunately “*S. islandicus”* is not a validly named species of the Archaea because it has not been validly named according to the International Code of Nomenclature of Bacteria (Lapage et al. 1992; Wayne 1987). As a result, no type strain exists for this “species”. Nonetheless, it is perhaps the most thoroughly studied prokaryotic organism from the standpoint of understanding its geographical distribution and several genomes of this species have been sequenced and annotated (Reno et al. 2009).

This investigation provides information on the inter-relatedness among the genomes of this organism.

## Materials and methods

Genomic sequences of the ten “*S. islandicus”* strains were downloaded from the NCBI FTP site (ftp://ncbi.nih.gov/genomes/Bacteria). Their accession numbers are NC_012588, NC_012589, NC_012622, NC_012623, NC_012632, NC_012726, NC_013769, NC_017275, NC_017276, and NC_021058. To infer phylogenetic relationships among these strains we used the whole-genome based and alignment-free CVTree method (Qi et al. 2004; Xu and Hao 2009). This method has high resolution at the strain level (Hao 2011) and does not require the identification of homologous proteins. The CVTrees were constructed for all available prokaryotic genomes using different peptide lengths from *K* = 3–7. Because the most reliable trees are obtained at *K* = 5 and 6 (Li et al. 2010), we only show the *K* = 6 data in this report.

For in silico DNA–DNA hybridization the sequences were submitted to the Genome-to-Genome Distance Calculator (GGDC) at DSMZ (Auch et al. 2010a, b ; DSMZ 2013). The program GGDC 2.0 was used and the most stringent distance function was chosen for the DDH values listed in Table 1. These values have been shown to have high correlation with the 16S rRNA distance and experimentally derived DDH values (Meier-Kolthoff et al. 2013). Another whole-genome-derived parameter, Average Nucleotide Identity (ANI), has been proposed as an alternative to experimental DDH values (Goris et al. 2007). We used the JSpecies software (Richter and Roselló-Móra 2009) to calculate ANI for the ten “*S. islandicus”* genomes.

**Table 1:** DDH values (in %) between “*S. islandicus”* strain pairs

	LAL	REY	HVE	M14	M162	M164	YN	YG	LS	LD
LAL		**92.9**	**88.9**	79.8	76.6	78.9	75.5	76.3	76.1	79.7
REY			**90.0**	82.7	78.6	81.7	78.3	79.6	81.2	80.7
HVE				83.0	79.5	83.3	79.4	76.7	81.8	78.3
M14					**95.4**	**93.6**	82.8	80.0	84.2	80.4
M162						**90.8**	79.8	75.8	79.8	76.0
M164							82.2	79.2	85.1	81.9
YN								**88.5**	85.8	82.4
YG									86.7	85.6
LS										**89.0**
LD										

Values between closest strains are provided in boldface

## Results and discussion

**Fig. 1:**
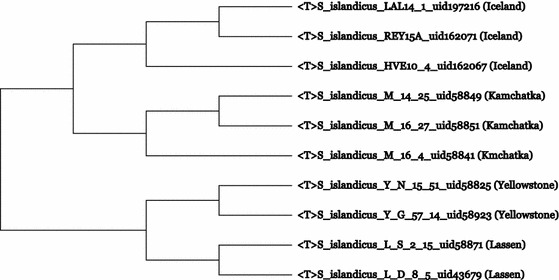
The whole genome CVTree of ten strains of “*S. islandicus”* based on 152 Archaea + 2286 Bacteria + 8 Eukarya at *K* = 6. The geographical origin of strains is given in *parentheses* at the end of each entry

Rachel Whitaker’s lab has studied the biogeographical distribution of seven “*S. islandicus”* strains isolated from two major continental locations, Euroasia (Iceland and Kamchatka, Russia) and North America (Yellowstone National Park, WY and Lassen National Park, CA) (Whitaker et al. 2003; Reno et al. 2009). Using MLSA, as well as whole genome analyses, a clear branching pattern of the phylogenetic tree according to the geographical separation of the strains was found. Subsequently, the genomes of three more “*S. islandicus”* strains were sequenced and analyzed (Guo et al. 2011; Jaubert et al. 2013). In whole-genome based CVTrees (Xu and Hao 2009) the phylogeny of these 10 strains is the same (Fig. 1) as that shown in Fig. 2 of Reno et al. (2009).

**Fig. 2:**
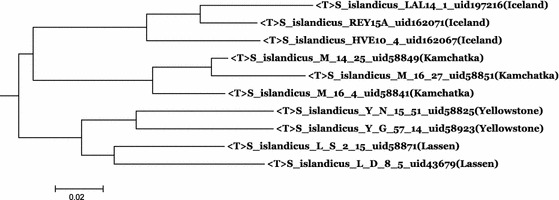
Tree constructed using distances calculated from the DDH values. Note that the 0.02 *bar* at the* lower-left corner* cannot be simply interpreted as “number of substitutions”, but it may indirectly reflect the evolutionary time span

Therefore the geographical pattern of the distribution of the strains is confirmed. However, the question remains: are these strains members of the same species? To assess this, DNA–DNA hybridization was calculated based on genomic sequences. Since genomes of these ten strains are available, it is unnecessary to conduct experiment determinations because in silico DDH tests are now available (Auch et al. 2010a, b). Furthermore, a public GGDC web site is provided for this analysis by DSMZ (2013).

The results of the pairwise DDH percentages for all ten strains of “*S. islandicus”* that were obtained by in silico DDH using the GGDC web server are shown in Table 1. These data indicate that the range of DDH of the strains within a particular location support their being members of the same species. For example, the strains from Icelandic hot springs range from 88.9 to 92.9 %. Strains from Russia show values from 90.8 to 95.4 %. Strains from North America range from 82.4 to 89.0 %. Since all of these values are substantially above the 70 % threshold defined by (Wayne 1987), it can be concluded that the strains sequenced from each location are all members of the same species.

The differences between locations are more marked, as one would expect based on the geographical separation among the groups of strains. The DDH values of the Icelandic strains compared to those from Kamchatka range from 76.6 to 83.3 % whereas their DDH values in comparison with the North American strains range from 75.5 to 81.8 %. The DDH values of the strains from Kamchatka compared to those from North American range from 75.8 to 85.1 %.

Therefore, using the currently accepted definition for prokaryote species, all of these strains, regardless of location, are members of the same species, “*S. islandicus”.* However, it is also clear that each geographic group comprises a separate variety or geovar (Staley and Gosink 1999). Indeed, some of the lowest DDH values found between strains from the different locations (75.5–76.6 %) are close to the threshold value of 70 %.

Using the DDH values, a tree can be constructed that shows the relatedness among the ten strains (Fig. 2). Here we define the “distance” between two strains using the following distance formula:

1 $${\rm Dis} = (100 - {\rm DDH})/100. $$

The topology of the tree derived from DDH values is noted to match that obtained in the whole genome CVTree (Fig. 1). It is notable from these trees and Table 1 that strains from the USA are more closely related than those from Iceland and Russia.

The ANI values calculated using the JSpecies software are given in Table 2. These values agree well but show slightly less divergence as compared to the DDH values in Table 1.

**Table 2:** ANI values (in %) between ‘*S. islandicus’* strain pairs

	LAL	REY	HVE	M14	M162	M164	YN	YG	LS	LD
LAL	**–**	**99.3**	**99.2**	98.3	98.4	98.3	98.3	98.4	98.4	98.2
REY	**99.3**	**–**	**99.4**	98.5	98.4	98.5	98.6	98.5	98.3	98.5
HVE	**99.2**	**99.3**	**–**	98.5	98.4	98.6	98.5	98.5	98.5	98.3
M14	98.0	98.3	98.5	**–**	**99.4**	**99.4**	98.2	98.2	98.2	98.3
M162	98.0	98.2	98.2	**99.3**	**–**	**99.3**	98.1	98.1	98.0	98.0
M164	98.3	98.6	98.6	**99.5**	**99.4**	**–**	98.5	98.5	98.5	98.4
YN	97.0	98.2	98.1	97.6	97.6	97.9	**–**	**99.3**	98.7	98.5
YG	98.2	98.3	98.3	97.9	98.0	98.1	**99.4**	**–**	98.9	98.9
LS	98.0	98.1	98.3	97.9	97.8	98.2	99.0	99.0	**–**	**99.2**
LD	97.7	98.0	97.9	98.0	97.7	98.0	98.7	98.7	**99.0**	**–**

Values between closest strains are provided in boldface. Due to the asymmetric relation between query and target in BLAST used in the intermediate stage of JSpecies there is no full off-diagonal symmetry in this table

Clearly our results support the view that biogeography plays a role in the speciation of “*S. islandicus”*. Further, an argument could be made that the strains at each of the locations should be defined as separate species because the current definition of a bacterial species is in flux and may be challenged (Krichevsky 2011; Richter and Roselló-Móra 2009; Staley 2006; Ward 1998).

At this time, in order to describe these strains as separate species, it is necessary to fulfill the criteria of the International Code for Nomenclature of Bacteria (Lapage et al. 1992; De Vos and Trüper 2000; Wayne 1987). Ideally, at least one significant phenotypic feature would need to be found that is unique for each proposed new species. One of the hypothetical questions this paper raises is: Would the geographic location of a strain suffice as an acceptable property for the description of a species? The source of a strain is already a primary feature used in the description of bacteria and archaea. However, until the discovery of the endemic biogeographical clustering of “*S. islandicus”* strains, it has not played a major role in the description of any species. Clearly more evidence would be needed before this property could be used as a primary property to separate one phylogenetically related geographic cluster from one area as a separate species, from a cluster in another area.

Also, it would be essential for the description of these geographic clusters as separate species that a ‘type strain’ from each area would need to be deposited in at least two different internationally accepted culture collections. As such, the authors recommend that those who work with “*S. islandicus”* follow the Interational Code of Nomenclature of Bacteria and provide cultures to culture collections so that this species can be validly named and appropriate strains designated and deposited to make them available for others to study.

## References

[CR1] Auch AF, Von Jan M, Klenk H-P, Gröker M (2010). Digital DNA–DNA hybridization for microbial species delineation by means of genome-to-genome sequence comparison. Stand Genomic Sci.

[CR2] Auch AF, Klenk H-P, Gröker M (2010). Standard operating procedure for calculating genome-to-genome distance based on high-scoring sequence segments. Stand Genomic Sci.

[CR3] Brenner DJ, Staley JT, Krieg NR, Garrity G, Krieg N, Brenner D, Staley JT (2005). Classification of procaryotic organisms and the concept of bacterial speciation. Bergey’s manual of systematic bacteriology.

[CR4] De Vos P, Trüper HG (2000). Judicial Commission of the International Committee on Systematic bacteriology. IXth International (IUMS) Congress of Bacteriology and Applied Microbiology. Minutes of the meetings, 14, 15 and 18 August 1999, Sydney, Australia. Int J Syst Evol Microbiol.

[CR5] DSMZ (2013) Deutsche sammlung von mikroorganismen und zellkulturen GGDC. http://ggdc.dsmz.de. Accessed 29 Aug 2013

[CR6] Goris J, Knostantinidis KT, Klappenbach JA (2007). DNA–DNA hybridization values and their relationship to whole-genome sequence similarities. Int J Syst Evol Microbiol.

[CR7] Guo L, Bruegger K, Liu C, Shah SA, Zheng H, Zhu Y, Wang S, Lillestol RK, Chen L, Frank J, Prangishvili D, Pulin L, She Q, Huang L, Garett RA (2011). Genome analysis of Icelandic strains of *Sulfolobus islandicus*: model organisms for genetic and virus–host interaction studies. J Bacteriol.

[CR8] Hao B (2011). CVTrees support the Bergey’s systematics and provide high resolution at species levels and below. Bull BISMiS.

[CR9] Jaubert C, Danioux C, Oberto J, Cortez D, Bize A, Krupovic M, She Q, Forterre P, Prangishvili D, Sezonov G (2013). Genomics and genetics of *Sulfolobus islandicus* LAL14/1, a model hyperthermophilic archaeon. Open Biol.

[CR10] Krichevsky MI (2011). What is a bacterial species? I will know it when I see it. Bull BISMiS.

[CR11] Lapage SP, Sneath PHA, Lessal EF (1992). International code of nomenclature of bacteria: bacteriological code 1990.

[CR12] Li Q, Xu Z, Hao B (2010). Composition vector approach to whole-genome-based prokaryotic phylogeny: success and foundations. J Biotechnol.

[CR13] Meier-Kolthoff JP, Auch AF, Klenk H-P, Göker M (2013). Genome sequence based species delineation with confidence intervals and improved distance functions. BMC Bioinformatics.

[CR14] Qi J, Wang B, Hao B (2004). Whole proteome prokaryote phylogeny without sequence alignment: a K-string composition approach. J Mol Evol.

[CR15] Reno ML, Held NL, Fields CJ, Burke PV, Whitaker RJ (2009). Biogeography of the *Sulfolobus islandicus* pan-genome. Proc Natl Acad Sci USA.

[CR16] Richter M, Roselló-Móra R (2009). Shifting the gold standard for the prokaryote species definition. Proc Natl Acad Sci USA.

[CR17] Staley JT, Bull AT (2004). Speciation and bacterial phylospecies. Microbial diversity and bioprospecting.

[CR18] Staley JT (2006). The bacterial species dilemma and the genomic-phylogenetic species concept. Phil Trans R Soc B.

[CR19] Staley JT, Gosink JJ (1999). Poles apart: biodiversity and biogeography of polar sea ice bacteria. Annu Rev Microbiol.

[CR20] Ward DM (1998). A natural species concept for prokaryotes. Curr Opin Microbiol.

[CR21] Wayne LG (1987). Report of the ad hoc committee on reconcilliation of approaches to bacterial systematics. Int J Syst Bacteriol.

[CR22] Whitaker RJ, Grogan DW, Taylor JW (2003). Geographic barrier isolate endemic populations of hyperthermophilic archaea. Science.

[CR23] Xu Z, Hao B (2009) CVTree update: a newly designed phylogenetic study platform using composition vectors and whole genomes. Nucl Acids Res 37 (Web Server Issue): W174–W178. The new version to be published in 2014 was used: http://tlife.fudan.edu.cn/cvtree3/. Accessed 5 July 2013 10.1093/nar/gkp278 PMC2703908 19398429

